# Soft-body dynamics induces energy efficiency in undulatory swimming: A deep learning study

**DOI:** 10.3389/frobt.2023.1102854

**Published:** 2023-02-09

**Authors:** Guanda Li, Jun Shintake, Mitsuhiro Hayashibe

**Affiliations:** ^1^ Neuro-Robotics Lab, Department of Robotics, Graduate School of Engineering, Tohoku University, Sendai, Japan; ^2^ Department of Mechanical and Intelligent Systems Engineering, The University of Electro-Communications, Chofu, Japan

**Keywords:** soft robot, energy efficiency, underwater robot, snake robot, deep reinforcement learning

## Abstract

Recently, soft robotics has gained considerable attention as it promises numerous applications thanks to unique features originating from the physical compliance of the robots. Biomimetic underwater robots are a promising application in soft robotics and are expected to achieve efficient swimming comparable to the real aquatic life in nature. However, the energy efficiency of soft robots of this type has not gained much attention and has been fully investigated previously. This paper presents a comparative study to verify the effect of soft-body dynamics on energy efficiency in underwater locomotion by comparing the swimming of soft and rigid snake robots. These robots have the same motor capacity, mass, and body dimensions while maintaining the same actuation degrees of freedom. Different gait patterns are explored using a controller based on grid search and the deep reinforcement learning controller to cover the large solution space for the actuation space. The quantitative analysis of the energy consumption of these gaits indicates that the soft snake robot consumed less energy to reach the same velocity as the rigid snake robot. When the robots swim at the same average velocity of 0.024 m/s, the required power for the soft-body robot is reduced by 80.4% compared to the rigid counterpart. The present study is expected to contribute to promoting a new research direction to emphasize the energy efficiency advantage of soft-body dynamics in robot design.

## 1 Introduction

Soft robotics, which is an emerging scientific field creating robots based on compliant materials, promises numerous applications such as object manipulation and human-robot interaction in industry, search and rescue activities in the natural environment, and rehabilitation in the medical Rus and Tolley, 2015; Rich et al., 2018; Shintake et al., 2018; Cianchetti et al., 2018; Jumet et al., 2022. Compared to traditional “rigid” robots, the compliant body of the soft robots is said to have better adaptability to the surrounding environment Rich et al., 2018; Aracri et al., 2021.

In this context, underwater soft robots, especially those based on biomimetics, are one of the promising applications in soft robotics Aracri et al., 2021. These biomimetic underwater robots exploit active deformations of their continuum body made of soft actuators. They have morphologies similar to those of their natural counterparts, such as fish Hubbard et al., 2013; Katzschmann et al., 2018; Shintake et al., 2018, snake Christianson et al., 2018; Nguyen and Ho, 2022, jellyfish Villanueva et al., 2011; Frame et al., 2018; Cheng et al., 2018; Ren et al., 2019, ray Park et al., 2016; Li et al., 2017, Li et al., 2021a, and flagellate Armanini et al., 2021.

Biomimetic soft underwater robots mimic the motion of aquatic animals with the expectation to achieve efficient swimming observed in nature. In this sense, compared to rigid underwater robots, the continuous deformation of the soft robots is expected to significantly improve the energy efficiency of swimming locomotion. Evidence suggests that the energy efficiency of soft-bodied robots improves with an augmented propulsive force due to fluid-inertial effects, as demonstrated in Giorgio-Serchi and Weymouth (2017). However, there has been no quantitative comparative study on the energy efficiency between soft and rigid robots under the constraints of having the same mass, size, shape, and input.

In order to shed light on the problem, in this study we investigate the effect of compliance on the energy efficiency in a specific swimming mode through a control experiment performed in a simulation environment. Different aspects can be considered for the simulation. These include, for instance, size, shape, weight, type of actuator, and material and mechanical properties. As the first comparative study, we focus on the dynamics of underwater locomotion as the main aspect. For the swimming mode, we employ anguilliform, a swimming mode enabled by the undulation of the snake-like slender body Sfakiotakis et al., 1999. The thin structure of the slender body is expected to simplify the simulation model and subsequent analysis. We compare the energy efficiency between soft-body and rigid-body snake robots with the same mass, size, shape, and motor capacity.

**FIGURE 1:**
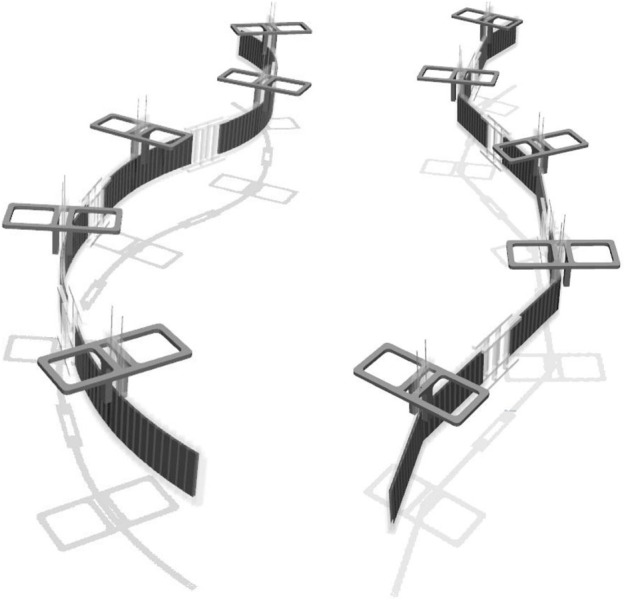
Morphological structures of a soft snake robot (left) and a rigid snake robot (right) in the simulation environment.

To precisely model the actuation and subsequent deformation of the body, we employ dielectric elastomer actuators (DEAs), due to their thin feature applicable to anguilliform swimming Christianson et al., 2018; Li et al., 2021b. Accordingly, we designed a soft snake robot and a rigid snake robot in a physical simulation environment, as shown in Figure 1.

The frequency at which the elasticity of the actuator oscillates plays a significant role in determining its overall efficiency. There is a significant body of evidence that suggests that the maximum level of efficiency is achieved when the actuator is operating at its natural resonant frequency Bujard et al., 2021; Zhong et al., 2021; Zheng et al., 2022. This highlights the importance of the elasticity of the actuator in terms of energy efficiency. However, one of the challenges in utilizing this principle is the difficulty in accurately determining the natural frequency of soft actuators. To address this issue, our work employs a combination of deep reinforcement learning and grid search methods to identify the most energy-efficient gait for snake robots. Through the use of these advanced techniques, we are able to overcome the limitations of traditional methods and make sure we can find the optimized solution for both the soft and rigid snake robots.

## 2 Simulation method

Current simulation methods for soft robots are limited, particularly for soft snake underwater robots composed of multiple soft actuators. The coupling effect between actuators, segments, and the surrounding water makes it more difficult to simulate underwater snake robots. Researchers have provided a method for underwater soft-robot simulation in the Du et al., 2021. However, the proposed method has only been validated on one degree of freedom (DoF) robot and requires the use of a real robot to collect data, which does not match the requirements of our experiments.

In our previous study Li et al., 2021b, we proposed an approximate simulation method for soft robot underwater locomotion in MuJoCo. MuJoCo is a physics engine that simulates multi-joint dynamics with contact and is widely used in robotics and biomechanics Todorov et al., 2012. In the previous study, we confirmed the accuracy of this simulation method by comparing a simulated robot with a real DEA fish robot. The result showed that the simulated soft robot had almost the same dynamics as a real soft robot.

**FIGURE 2:**
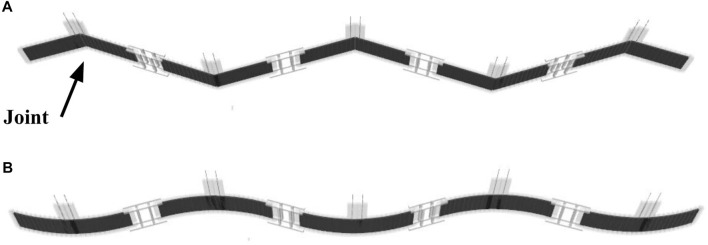
The simulation model of snake robots in MuJoCo. **(A)** The rigid snake robot. **(B)** The soft snake robot. One DoF per segment for both models.

As such, in this study, we use MuJoCo to design the soft and rigid snake robots with the same size (length 27.0 cm, width 1.5 cm, and thickness 0.5 cm), mass (7.93 g), and output torque of actuators (12 Nm). The models of these robots are shown in Figure 2. The soft snake robot has five segments, every of which actively deforms independently with the simulated actuation behavior of a DEA. Each segment is split into a connected series of thin-sliced elements to realize the deformable attribution of the DEA, which are alternately connected by active joints and passive joints in the rotation. The active joints were controlled by the output torque of the simulated motor set on it, which was used to simulate the output characteristics of the DEA. All active joints in one segment are simultaneously controlled by the same input signal. The characteristics of the DEAs can be adjusted by tuning the attributes of passive joints.

There are 12 active and 11 passive joints per segment for the soft-body case. However, the actuation is still one DoF per segment as the shared same control input is applied to the active joints. One segment has only one DoF, resulting in curved body deformation. Since the soft snake robot has five segments, there are five DoFs in total for the actuation space. The range of rotation of the active joints is set to [−3°, 3°]. The output torque range of the active joint is set to [−1, 1] Nm.

The rigid snake robot has six segments with five joints, as shown in Figure 2. A simulated motor is placed on each of the five joints. Each segment has one DoF by bending at the joint. In total, there are five DoFs for the actuation space, identical to the soft snake robot. The robot is controlled by changing the output torque of the motors. To ensure a fair comparison, the actuation range of the rigid snake robot’s joints was set to [−36°, 36°] to match the total actuation range of the soft snake robot, which has 12 active joints per segment, each with an actuation range of [−3°, 3°] (−36°, 36° in total). The range of the output torque of the active joint is set to [−12, 12] Nm. Twelve times more torque capacity is set for the joint torque for the rigid robot to obtain the equivalent torque capacity of the soft robot, which has 12 distributed actuators with a 1 Nm torque range.

The density and viscosity of the liquid in the simulation environment are set to 1,000 kg/m^3^ and 0.0009 Pa⋅ s, respectively. The simulated frequency is 100 Hz, and the control frequency is 50 Hz.

## 3 Controller design

We present two methods for controlling the underwater locomotion of snake robots. The methods are used to find the gaits of snake robots in water; then, the average velocity and the required average power are verified to evaluate the energy efficiency of the snake robot. The gait equation controller is model-based. It continuously generates joint torques for the snake robot according to a pre-defined equation. The DRL controller is data-based. During the training process of the DRL controller, the snake robot could explore different gaits beyond the limitations of the gait equation controller.

### 3.1 Gait equation controller

The gait equation controller we used to drive the snake robots is modelled as

$$\tau \left(n,t\right)=A\times \sin\left(\omega t+n\varphi \right),$$ (1)

where *τ*(*n*, *t*) represents the output torque of the motor on the *n*th joint of the snake robots at moment *t*. *A* denotes torque amplitude. *ω* and *ϕ* denote spatial and temporal frequencies, respectively.

We can make the snake robot swim in different gaits by changing the values of *A*, *ω* and *ϕ*. Therefore, we used the grid search method to determine the optimal parameters of the gait equation controller. The parameters and interval ranges of the grid search are listed in Table 1. The selection of the parameters and interval ranges was done through a process of trial and error. The setting the amplitude or temporal frequency too high resulted in unstable simulations and too low interval range would increase the simulation time. We aimed to find the best balance between including a wide range of motion patterns and maintaining stability and efficiency in the simulation.

**TABLE 1:** The parameters for the grid search.

Paramaters	Values	Descriptions
*ω*	0.1, 0.6, 1.1, 1.6, 2.1, 2.6	Temporal frequency
	3.1, 3.6, 4.1, 4.6, 5.1, 5.6	
	6.1, 6.6, 7.1, 7.6, 8.1, 8.6	
*A*	0.1, 0.2, 0.3, 0.4, 0.5, 0.6	Amplitude
	0.7, 0.8, 0.9, 1.0, 1.1, 1.2	
	1.3, 1.4, 1.5, 1.6, 1.7, 1.8	
	1.9, 2.0	
*ϕ*	18, 36, 54, 72, 90, 108	Spatial frequency (in degrees)
	126, 144, 162, 180	

### 3.2 DRL controller

The DRL controller is a neural network controller trained by the deep reinforcement learning algorithm. When using this method, robots learn the target skill through interaction with the environment Mnih et al., 2013; Arulkumaran et al., 2017 In this process, the robot collects a large number of state-action pairs to evaluate the data according to the reward function. Through continuous iterative training, the robot can discover better state-action pairs until training converges.

The state is represented in the data collected by the robot from the environment, such as the robot’s joint position and angular velocity, which correspond to the input layer of the neural network. Action is the command used to drive the robot actuators and corresponds to the output layer of the neural network.

Deep reinforcement learning is primarily used in robotics for locomotion control Jangir et al., 2020; Lee et al., 2020 and navigation Fan et al., 2020; Zeng et al., 2021. When a robot is trained for locomotion, it can steadily find synergetic motor action patterns similar to those of animals Chai and Hayashibe, 2020. Deep reinforcement learning algorithms have successfully found better gaits for a rigid snake robot on land Bing et al., 2020.

Snake robots can explore more gaits that cannot be found by grid search. It is achieved by using the DRL controller as a model-free method to overcome the limitations of the established equations.

#### 3.2.1 Algorithm

The deep reinforcement learning algorithm we used to train the snake robots is proximal policy optimization (PPO) Schulman et al., 2017, which is an on-policy algorithm and is widely used to handle continuous action space tasks Mahmood et al., 2018.

#### 3.2.2 Reward function

In our experiments, we designed two different reward functions $R_{1}$ and $R_{2}$ , for the robot to learn as many different gaits as possible.

In the

$$R_{1}=\alpha Vel_{x}-\beta \underset{J}{\sum }\left|\tau_{j}\omega_{j}\right|\Delta t,$$ (2)

where *Vel*_*x*_ denotes the velocity of the center of mass of the snake robot along *x*-axis. The faster the snake robot moved in the positive direction of the *x*-axis, the larger the rewards the robot could receive.

The second term $\sum_{J}\left|\tau_{j}\omega_{j}\right|\Delta t$ is the energy consumed by the robot in one timestep Δ*t*. In our experiments, Δ*t* was 0.02 s. The energy consumed reduced the reward received by the robot. *α* and *β* are scaling factors used to adjust the magnitude of the rewards.

**FIGURE 3:**
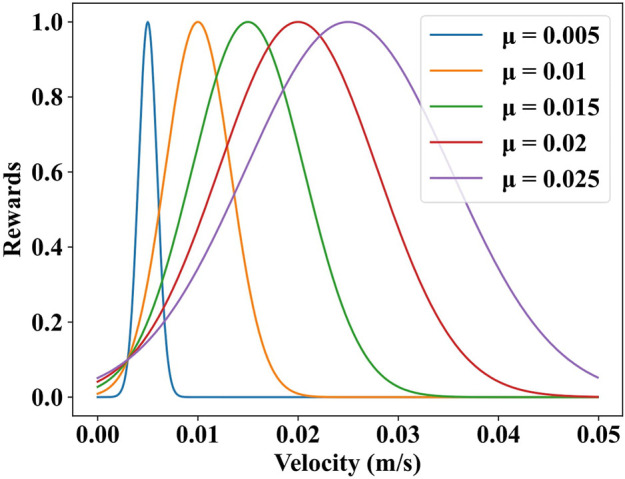
The relationship between velocity and reward. *μ* is the target velocity of snake robots. The position of the margin point is (0.003, 0.1).

In the

$$\begin{array}{cc} R_{2} & =\alpha f\left(Vel_{x};\mu ,\sigma \right)-\beta \underset{J}{\sum }\left|\tau_{j}\omega_{j}\right|\Delta t\\ f\left(Vel_{x};\mu ,\sigma \right) & =\frac{1}{\sigma \sqrt{2\pi }}\exp\left(-\frac{{\left(Vel_{x}-\mu \right)}^{2}}{2\sigma^{2}}\right), \end{array}$$ (3)

we set the target velocity of the snake robots using a Gaussian distribution *f* (*Vel*_*x*_; *μ*, *σ*), where *Vel*_*x*_ denotes the current velocity of the robot. *μ* denotes the target velocity of the robot, as shown in Figure 3. The other terms are the same as those in $R_{1}$ .

#### 3.2.3 Observation space

The observation space contains the information that the robot obtains from the environment to perform feedback control.

The observation space O used to train the snake robots is

$$\begin{array}{ll} O & =\left[Vel_{x1},Vel_{x2},Vel_{x3},Vel_{x4},Vel_{x5},\right.\\ & \;Vel_{y1},Vel_{y2},Vel_{y3},Vel_{y4},Vel_{y5},\\ & \;\left.Pos_{x1},Pos_{x2},Pos_{x3},Pos_{x4},Pos_{x5}\right]. \end{array}$$ (4)

It contains the displacements of the five segments on the *y*-axis, and the velocities on the x- and *y*-axis.

#### 3.2.4 Action space

Action space A has the dimensions of the number of actuators of the snake robot. Each element in the action space corresponds to an actuator in the robot. For both the soft and rigid snake robots, the size of the action space was five.

#### 3.2.5 Training configuration

We deployed our training in RLlib Liang et al., 2018, which is a distributed reinforcement learning framework that could allocate computing resources conveniently.

Furthermore, we used a two-layer fully connected network with 256 units per layer as the hidden layer of the policy network. The input layer of the policy network had the same dimension as the observation space O and the output layer had the same dimensions as the action space A .

We obtained two groups of training results by adjusting the values of *α* and *β* in the reward functions $R_{1}$ and $R_{2}$ . Three random seeds are used for each training to reduce the uncertainty of the results.

## 4 Results and analysis

We compare and analyze the differences in energy consumption between the gait equation controllers and the deep reinforcement learning controllers. We determined the energy efficiency of the snake robots by comparing the relationship between the average velocity of the CoM and the average output power required to drive the system. A gait with a higher average velocity at the same output power has better energy efficiency. In other words, the gait could be managed with less output power at the same motion velocity, it also demonstrated better energy efficiency.

We calculated the average velocity *V* and average output power *P* of all the actuators on the snake robots using the following two equations:

$$V=\frac{1}{T}\overset{T}{\underset{t=0}{\sum }}\sqrt{{Vel_{x}\left(t\right)}^{2}+{Vel_{y}\left(t\right)}^{2}},$$ (5)

$$P=\frac{1}{T}\overset{T}{\underset{t=0}{\sum }}\underset{J}{\sum }\left|\tau_{j}\left(t\right)\omega_{j}\left(t\right)\right|\Delta t,$$ (6)

where *T* denotes the number of time steps in the simulation. The experiments described in this section had 1,000 timesteps (20 s) for each round of testing.

### 4.1 Results of gait equation controller

**FIGURE 4:**
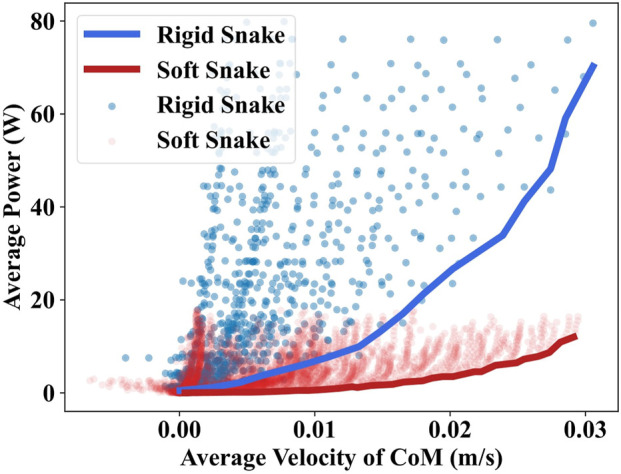
The grid search results of the gait equation controller for the soft and rigid snake robot. Each point in the scatter plot corresponds to a set of parameters in the grid equation controller. The fold line is the lower contour line of the same color scatter plot.

Utilizing the grid search, the gait equation controller generated 3,600 different gaits for the soft snake robot and rigid snake robot. In Figure 4, we present the results of the average velocity of the CoM and the average power of the two snake robots using scatter plots for a maximum speed of less than 0.03 m/s. The comparison of the lower contour lines of the two results shows that the soft snake robot requires significantly less energy than the rigid snake robot to reach the same velocity, and the gap in energy consumption is further magnified as the velocity increases.

### 4.2 Results of deep reinforcement learning

**FIGURE 5:**
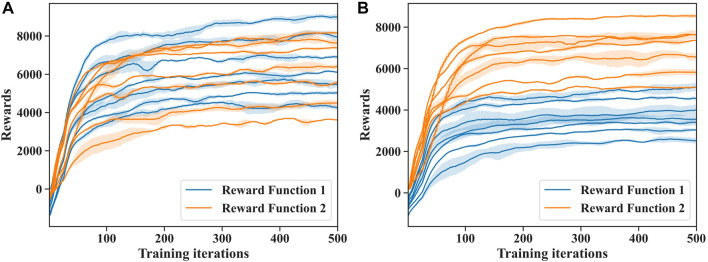
**(A)** Deep reinforcement learning training progress by two different reward functions for soft snake robot underwater locomotion. **(B)** Deep reinforcement learning training progress by two different reward functions for rigid snake robot underwater locomotion.

In Figure 5, we illustrate the process of training DRL controllers for the rigid snake robot and soft snake robot using the two different reward functions $R_{1}$ and $R_{2}$ mentioned in the previous section. The figures present the training results of the two reward functions with the seven groups of parameters. The solid line and shaded part represent the mean and variance of the training results with different random seeds. As the number of training iterations increases, the policy network can obtain higher rewards in each round of testing, indicating that the snake robot can accomplish the tasks expected from the reward functions. The training results converged after 500 iterations. We used the neural network at the endpoint as the DRL controller to evaluate the energy efficiency of the soft and rigid snake robots.

### 4.3 Comparison

**FIGURE 6:**
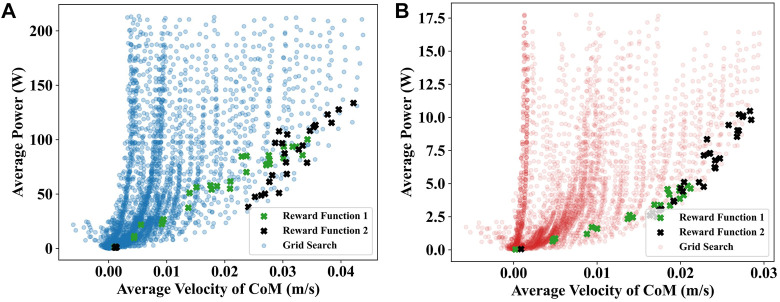
**(A)** Comparision of the testing result of deep reinforcement learning controllers and gait equation controllers for rigid snake robot. **(B)** Comparision of the testing result of deep reinforcement learning controllers and gait equation controllers for soft snake robot. The plots with reward functions are corresponding to the deep learning solutions.

In Figure 6, we compare the test results of the gait equation controller and DRL controller of the snake robots using scatter plots. The test results demonstrate that almost all of the gaits generated by the DRL controller are within the range of the gait equation controller’s results indicating that the gait equation controller using grid search with very narrow intervals can provide a sufficient exploration of potential action patterns. Moreover, the results also demonstrate that the deep learning solutions are well distributed around the lower bottom side of the average power for Figure 6. It is interesting to confirm that deep learning works well for exploring the swimming solution space. Noticeably, Reward Function $R_{2}$ is suitable for finding faster swimming patterns, and Reward Function $R_{1}$ is suited for lower energy consumption with slower swimming patterns.

Special attention should be given to the DRL controllers of the soft snake robot. The results were much closer to the boundary of the gait equation test results than in the rigid snake robot case. In other words, in the same training situation, it is more challenging for rigid snake robot to learn energy-efficient gaits using DRL. This is a further evidence suggests that the soft snake robot’s physical attributes make it more energy-efficient than the rigid robot when moving underwater.

In Section II, we introduced the range of the joint rotation and output torque of the rigid snake robot which is calculated based on the joint rotation range, output torque, and the number of active joints of the soft snake robot. We changed the joint rotation range and output range of the rigid snake robot to exclude the potential effects of the joint range and output torque range on the experimental results and performed additional comparative experiments.

**FIGURE 7:**
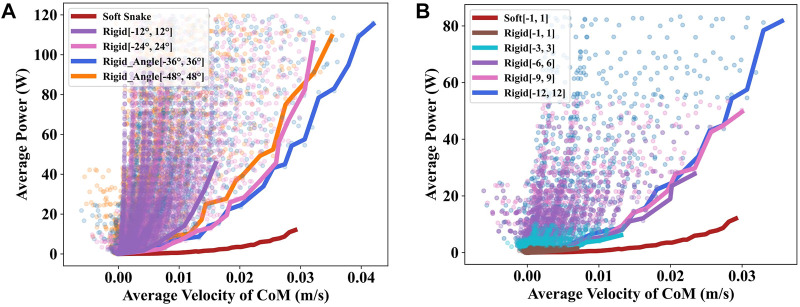
**(A)** Comparision of different rotation ranges of rigid snake robot’s motor. **(B)** Comparision of different output torque ranges of rigid snake robot’s motor.

First, we change the joint rotation range of the rigid snake robot. The original joint angle rotation range was [−36°, 36°]. Here we changed it to [−12°, 12°], [−24°, 24°], and [−48°, 48°] and performed the same test separately. The experimental results in Figure 7A indicate that the original range of rotation exhibits the best performance among the four sets of values. Generally, an excessively large or small rotation angle range will cause more energy consumption. Then we changed the output torque range of the rigid snake robot’s actuators from [−12, 12] Nm to [−1, 1] Nm, [−3, 3] Nm, [−6, 6] Nm, and [−9, 9] Nm. In the results shown in Figure 7B, the lower contour lines of the scatter plots for different torque ranges exhibit a very similar growing trend.

The results of the smaller torque range are overlaid by those of the larger torque range. It is primarily because when we perform the grid search for the gait equation controller, the search range of the torque amplitude A is [0.1, 2] Nm with a 0.1 search interval, and the original search result already contains part of the result of a smaller torque range.

Since the other parameters of the grid search were unchanged when we performed these experiments, the fact that no better gaits were found implies we have already fully explored all the gaits that can be generated by the gait equation.

### 4.4 Analysis

Based on the above experimental results, the soft robot can use the advantages of its distinct deformed body to achieve better energy efficiency when moving underwater.

**FIGURE 8:**
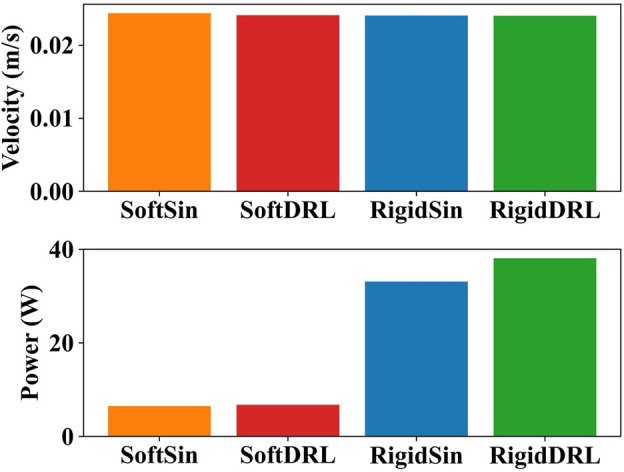
The test results for the four selected gaits, in order from left to right, are soft snake robot with gait equation controller, soft snake robot with DRL controller, rigid snake robot with gait equation controller and rigid snake robot with DRL controller.

To analyze the reasons for this difference, four test results with similar average velocity and lowest output power were selected for the soft and rigid snake robots, respectively, for comparison. These results were individually generated using the DRL and gait equation controller, as shown in Figure 8. We calculated the results of the gait equation controller and found that when the snake robot had the same average velocity of 0.024 m/s, the output power of the soft-body snake robot was only 19.60% of that of the rigid-body snake robot.

**FIGURE 9:**
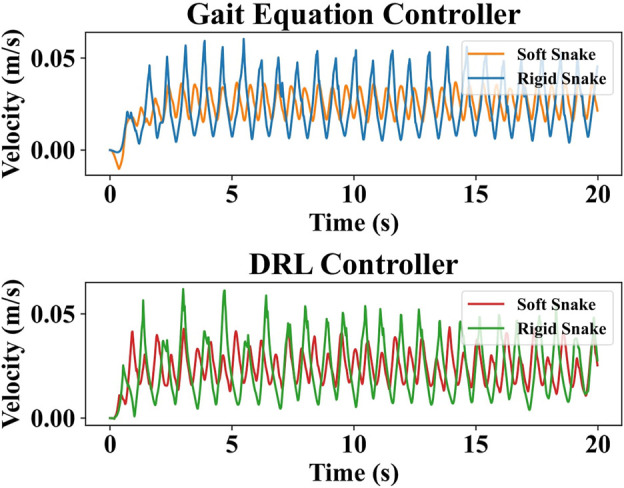
Comparison of the center-of-mass velocities of the four gaits.

In Figure 9, we plot the variation in the CoM velocity of the snake robot in these four gaits. The results indicate that the rigid snake robot has greater variations in CoM velocity as it swims, which is an underlying reason behind its low energy efficiency. The larger velocity variations imply the situation where the robot body has the larger drag force against the water flow.

**FIGURE 10:**
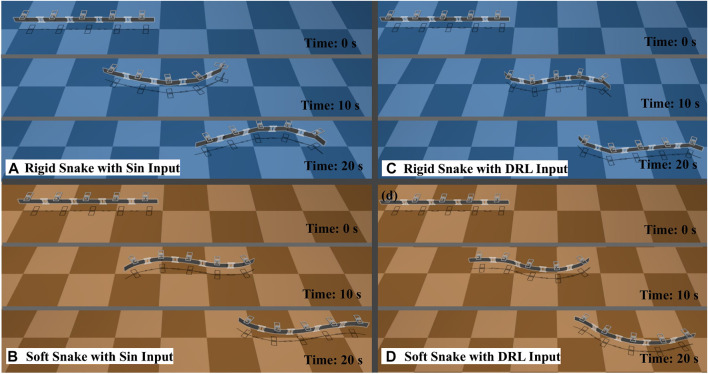
Test results for the four selected gaits. **(A)** The rigid snake robot is controlled by the gait equation controller. **(B)** The rigid snake robot is controlled by the DRL controller. **(C)** The soft snake robot is controlled by the gait equation controller. **(D)** The soft snake robot is controlled by the DRL controller.

The reason for having different drag forces is the continuous shape of the soft body robot can provide a smoother gradient of the body shape, which can significantly reduce the drag from the water. As the soft robot swimming, it can efficiently transport the surrounding water backwards while minimizing the drag, as shown in Figure 10.

## 5 Conclusion

We demonstrated that soft-body induces swimming motions to have better energy efficiency when moving underwater by comparing the different gaits of soft and rigid snake robots. First, we designed a soft snake robot and a rigid snake robot with the same dimensions, mass, and actuation power in a simulation environment while keeping the same degrees of freedom for the actuation space. Subsequently, we determined the optimal energy utilization gaits of the two robots explored by grid search and deep reinforcement learning methods for covering the large solution space for the control inputs. By comparing these gaits, we discovered that the average output power required by the soft-body robot is much smaller than that required by the rigid-body robot when it reached the same speed. The main reason for this is that the soft robot has less velocity variation of the body when moving underwater and the continuous body shape of the soft robot reduces the drag from the water. Then the soft robot can effectively transport the surrounding water backwards while minimizing the drag. Our work confirms the advantages of soft-body dynamics against rigid-body dynamics in snake-like underwater robots. We believe that this study can contribute to promoting a new direction to emphasize the energy efficiency advantage of soft-body dynamics for robot design. In the future, we will further analyze the advantages of soft-body dynamics for energy-efficient motion generation by comparing different motor tasks, both underwater and on the ground.

## Data Availability

The raw data supporting the conclusion of this article will be made available by the authors, without undue reservation.

## References

[B1] Aracri S. Giorgio-Serchi F. Suaria G. Sayed M. E. Nemitz M. P. Mahon S. (2021). Soft robots for ocean exploration and offshore operations: A perspective. Soft Robot. 8, 625–639. 10.1089/soro.2020.0011 33450174 PMC8713554

[B2] Armanini C. Farman M. Calisti M. Giorgio-Serchi F. Stefanini C. Renda F. (2021). Flagellate underwater robotics at macroscale: Design, modeling, and characterization. IEEE Trans. Robotics 38, 731–747. 10.1109/tro.2021.3094051

[B3] Arulkumaran K. Deisenroth M. P. Brundage M. Bharath A. A. (2017). Deep reinforcement learning: A brief survey. IEEE Signal Process. Mag. 34, 26–38. 10.1109/msp.2017.2743240

[B4] Bing Z. Lemke C. Cheng L. Huang K. Knoll A. (2020). Energy-efficient and damage-recovery slithering gait design for a snake-like robot based on reinforcement learning and inverse reinforcement learning. Neural Netw. 129, 323–333. 10.1016/j.neunet.2020.05.029 32593929

[B5] Bujard T. Giorgio-Serchi F. Weymouth G. D. (2021). A resonant squid-inspired robot unlocks biological propulsive efficiency. Sci. Robotics 6, eabd2971. 10.1126/scirobotics.abd2971 34043579

[B6] Chai J. Hayashibe M. (2020). Motor synergy development in high-performing deep reinforcement learning algorithms. IEEE Robotics Automation Lett. 5, 1271–1278. 10.1109/lra.2020.2968067

[B7] Cheng T. Li G. Liang Y. Zhang M. Liu B. Wong T.-W. (2018). Untethered soft robotic jellyfish. Smart Mater. Struct. 28, 015019. 10.1088/1361-665x/aaed4f

[B8] Christianson C. Goldberg N. N. Deheyn D. D. Cai S. Tolley M. T. (2018). Translucent soft robots driven by frameless fluid electrode dielectric elastomer actuators. Sci. Robotics 3, eaat1893. 10.1126/scirobotics.aat1893 33141742

[B9] Cianchetti M. Laschi C. Menciassi A. Dario P. (2018). Biomedical applications of soft robotics. Nat. Rev. Mater. 3, 143–153. 10.1038/s41578-018-0022-y

[B10] Du T. Hughes J. Wah S. Matusik W. Rus D. (2021). Underwater soft robot modeling and control with differentiable simulation. IEEE Robotics Automation Lett. 6, 4994–5001. 10.1109/lra.2021.3070305

[B11] Fan T. Long P. Liu W. Pan J. (2020). Distributed multi-robot collision avoidance via deep reinforcement learning for navigation in complex scenarios. Int. J. Robotics Res. 39, 856–892. 10.1177/0278364920916531

[B12] Frame J. Lopez N. Curet O. Engeberg E. D. (2018). Thrust force characterization of free-swimming soft robotic jellyfish. Bioinspiration biomimetics 13, 064001. 10.1088/1748-3190/aadcb3 30226216

[B13] Giorgio-Serchi F. Weymouth G. D. (2017). “Underwater soft robotics, the benefit of body-shape variations in aquatic propulsion,” in Soft robotics: Trends, applications and challenges (Springer), 37–46.

[B14] Hubbard J. J. Fleming M. Palmre V. Pugal D. Kim K. J. Leang K. K. (2013). Monolithic ipmc fins for propulsion and maneuvering in bioinspired underwater robotics. IEEE J. Ocean. Eng. 39, 540–551. 10.1109/joe.2013.2259318

[B15] Jangir R. Alenyà G. Torras C. (2020). “Dynamic cloth manipulation with deep reinforcement learning,” in 2020 IEEE international conference on robotics and automation (ICRA) (IEEE), 4630–4636.

[B16] Jumet B. Bell M. D. Sanchez V. Preston D. J. (2022). A data-driven review of soft robotics. Adv. Intell. Syst. 4, 2100163. 10.1002/aisy.202100163

[B17] Katzschmann R. K. DelPreto J. MacCurdy R. Rus D. (2018). Exploration of underwater life with an acoustically controlled soft robotic fish. Sci. Robotics 3, eaar3449. 10.1126/scirobotics.aar3449 33141748

[B18] Lee J. Hwangbo J. Wellhausen L. Koltun V. Hutter M. (2020). Learning quadrupedal locomotion over challenging terrain. Sci. robotics 5, eabc5986. 10.1126/scirobotics.abc5986 33087482

[B19] Li G. Chen X. Zhou F. Liang Y. Xiao Y. Cao X. (2021a). Self-powered soft robot in the mariana trench. Nature 591, 66–71. 10.1038/s41586-020-03153-z 33658693

[B20] Li G. Shintake J. Hayashibe M. (2021b). “Deep reinforcement learning framework for underwater locomotion of soft robot,” in 2021 IEEE international conference on robotics and automation (ICRA) (IEEE), 12033–12039.

[B21] Li T. Li G. Liang Y. Cheng T. Dai J. Yang X. (2017). Fast-moving soft electronic fish. Sci. Adv. 3, e1602045. 10.1126/sciadv.1602045 28435879 PMC5381956

[B22] Liang E. Liaw R. Nishihara R. Moritz P. Fox R. Goldberg K. (2018). “Rllib: Abstractions for distributed reinforcement learning,” in International conference on machine learning (Stockholm, Sweden: PMLR), 3053–3062.

[B23] Mahmood A. R. Korenkevych D. Vasan G. Ma W. Bergstra J. (2018). “Benchmarking reinforcement learning algorithms on real-world robots,” in Conference on robot learning (Zürich, Switzerland: PMLR), 561–591.

[B24] Mnih V. Kavukcuoglu K. Silver D. Graves A. Antonoglou I. Wierstra D. (2013). Playing atari with deep reinforcement learning. arXiv preprint arXiv:1312.5602.

[B25] Nguyen D. Q. Ho V. A. (2022). Anguilliform swimming performance of an eel-inspired soft robot. Soft Robot. 9, 425–439. 10.1089/soro.2020.0093 34134542

[B26] Park S.-J. Gazzola M. Park K. S. Park S. Di Santo V. Blevins E. L. (2016). Phototactic guidance of a tissue-engineered soft-robotic ray. Science 353, 158–162. 10.1126/science.aaf4292 27387948 PMC5526330

[B27] Ren Z. Hu W. Dong X. Sitti M. (2019). Multi-functional soft-bodied jellyfish-like swimming. Nat. Commun. 10, 2703–2712. 10.1038/s41467-019-10549-7 31266939 PMC6606650

[B28] Rich S. I. Wood R. J. Majidi C. (2018). Untethered soft robotics. Nat. Electron. 1, 102–112. 10.1038/s41928-018-0024-1

[B29] Rus D. Tolley M. T. (2015). Design, fabrication and control of soft robots. Nature 521, 467–475. 10.1038/nature14543 26017446

[B30] Schulman J. Wolski F. Dhariwal P. Radford A. Klimov O. (2017). Proximal policy optimization algorithms. arXiv preprint arXiv:1707.06347.

[B31] Sfakiotakis M. Lane D. M. Davies J. B. C. (1999). Review of fish swimming modes for aquatic locomotion. IEEE J. Ocean. Eng. 24, 237–252. 10.1109/48.757275

[B32] Shintake J. Cacucciolo V. Shea H. Floreano D. (2018). Soft biomimetic fish robot made of dielectric elastomer actuators. Soft Robot. 5, 466–474. 10.1089/soro.2017.0062 29957131 PMC6101101

[B33] Todorov E. Erez T. Tassa Y. (2012). “Mujoco: A physics engine for model-based control,” in 2012 IEEE/RSJ international conference on intelligent robots and systems (IEEE), 5026–5033.

[B34] Villanueva A. Smith C. Priya S. (2011). A biomimetic robotic jellyfish (robojelly) actuated by shape memory alloy composite actuators. Bioinspiration biomimetics 6, 036004. 10.1088/1748-3182/6/3/036004 21852714

[B35] Zeng Y. Xu X. Jin S. Zhang R. (2021). Simultaneous navigation and radio mapping for cellular-connected uav with deep reinforcement learning. IEEE Trans. Wirel. Commun. 20, 4205–4220. 10.1109/twc.2021.3056573

[B36] Zheng C. Li G. Hayashibe M. (2022). Joint elasticity produces energy efficiency in underwater locomotion: Verification with deep reinforcement learning. Front. Robotics AI 9, 957931. 10.3389/frobt.2022.957931 PMC9493006 36158602

[B37] Zhong Q. Zhu J. Fish F. E. Kerr S. J. Downs A. Bart-Smith H. (2021). Tunable stiffness enables fast and efficient swimming in fish-like robots. Sci. Robotics 6, eabe4088. 10.1126/scirobotics.abe4088 34380755

